# B-Cell Lymphoma Diagnosed in an Explanted Liver of a Patient With Metabolic Dysfunction–Associated Steatohepatitis Cirrhosis

**DOI:** 10.14309/crj.0000000000001656

**Published:** 2025-04-07

**Authors:** Lily Kaufman, Hannah Chi, Luke Bushrow, Martha Yearsley, Khalid Mumtaz

**Affiliations:** 1 Ohio State University, Wexner Medical Center, Columbus, OH

**Keywords:** explant, lymphoma, liver transplant, MASH, hepatic malignancy

## Abstract

Hepatic malignancies in explanted livers are not uncommon; however, finding primary nonhepatic malignancies in explanted livers of any etiology is very rare. Given its rarity, we present a case of low-grade B-cell lymphoma in the liver explant of a patient with metabolic dysfunction–associated steatohepatitis cirrhosis after orthotopic liver transplantation. Preorthotopic liver transplantation workup was notable for periportal lymphadenopathy that was negative for malignancy per ultrasound-guided biopsy, so this finding was surprising. This unexpected diagnosis of lymphoma despite negative workup during pretransplant evaluation underscores the importance for liver transplant centers to conduct thorough investigations for malignancies before transplantation.

## INTRODUCTION

Orthotopic liver transplantation (OLT) is the mainstay of treatment to improve life expectancy and quality of life for patients with decompensated hepatic cirrhosis.^1^ Candidates for OLT require extensive medical, social, and psychological workup, followed by evaluation of the explanted liver after transplantation to assess for concordance with pretransplant diagnosis.^2^ The discovery of incidental or unknown hepatic malignancy via explant pathology is not uncommon.^3^ Current literature has largely described cases of hepatic malignancy discovered on explanted livers in which the etiology of cirrhosis was alcohol abuse or viral hepatitis.^4,5^ However, diagnosis of primary nonliver malignancy in a liver explant is very rare.

Given its rarity, we report a case of a patient with decompensated metabolic dysfunction–associated steatohepatitis (MASH) cirrhosis who underwent OLT without preoperative diagnosis of a hepatic malignancy. To our surprise, explant liver pathology showed features of low-grade B-cell lymphoma.

## CASE REPORT

A 69-year-old man with decompensated MASH cirrhosis and model for end-stage liver disease 3.0 score of 21 underwent workup for OLT. His medical history included diabetes mellitus and hypothyroidism. His medications included insulin, spironolactone, levothyroxine, tamsulosin, and furosemide. Three months before OLT, CT abdomen with contrast demonstrated no suspicious liver lesions but was remarkable for bulky retroperitoneal and periportal lymphadenopathy. A comprehensive workup, including endoscopic ultrasound-guided biopsy of periportal lymph nodes, was negative for malignancy or lymphoma. Alpha-fetoprotein (AFP) level was less than 2.2 ng/mL during pretransplant workup, and CA 19-9 was not collected as there was no suspicion of cholangiocarcinoma. The patient's case was discussed at our institutional liver tumor board and patient selection committee, and it was advised that further malignancy workup was not needed to proceed with listing for transplantation.

He underwent OLT from a brain-dead donor, and the explanted liver was sent for histopathologic analysis. Intraoperative and post-OLT course was uncomplicated, and he was discharged on postoperative day 7. His discharge immunosuppressive medications were tacrolimus and mycophenolate mofetil. Labs taken before discharge showed target immunosuppression levels and downtrending liver enzymes.

**Figure 1.**
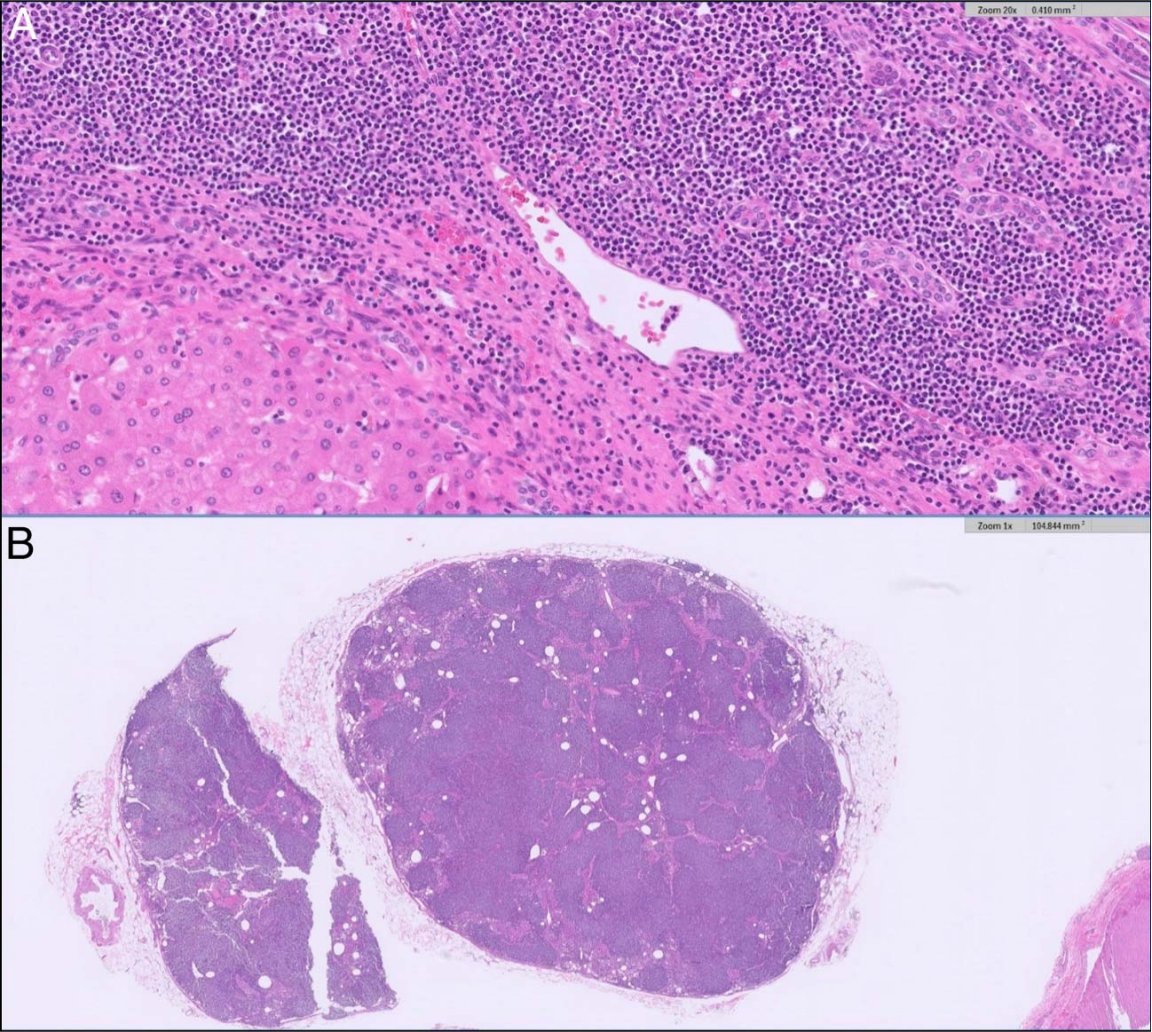
(A) Histopathology of the explanted liver. Dense septal lymphocytic infiltrate comprised predominantly small cells with variably irregular nuclei and condensed chromatin. (B) Histopathology of a hepatic lymph node. There is total effacement of the normal lymph node architecture by a diffuse lymphoid proliferation with cytomorphology similar to the hepatic infiltrate. Immunostains reveal the diffuse infiltrate to express C20 (shown below), CD5 (shown below), PAX5, and CD43.

Histopathologic analysis of the explanted liver showed features of MASH cirrhosis. Additional analysis also demonstrated dense septal lymphoid infiltrate with predominantly small cells with scant cytoplasm, round to slightly irregular nuclei, and condensed chromatin. The hepatic lymph nodes showed effacement of nodal architecture by a diffuse lymphoid proliferation, comprised lymphocytes with similar cytomorphology as those found in the liver explant. The B-cell population in the lymph nodes expressed Pax-5, CD20, CD5 (dim), and CD43 (dim) and was negative for CD10, SOX11, BCL-1, BCL-6, MUM1, and CD138. Molecular studies by IgH PCR detected the presence of a clonal B-cell population. A diagnosis of low-grade, CD5 dim B-cell lymphoma was made, a nonspecific phenotype that may be seen in chronic lymphocytic leukemia/small lymphocytic lymphoma and CD5^+^ marginal zone lymphoma (Figure 1).

A diagnosis of low-grade B-cell lymphoma was confirmed. FDG-PET scan was obtained, which was negative for obvious findings of lymphoma in the abdomen and chest. Hematology was consulted and suggested no additional treatment due to lack of evidence of active lymphoma on PET scan. The patient was advised a close follow-up strategy for disease surveillance of lymphoma via CT/PET scans every 3 months. During a follow-up clinic visit 2 months post-OLT, he was switched from tacrolimus to everolimus and maintained on a low dose of mycophenolate mofetil.

## DISCUSSION

We reported a rare case of low-grade B-cell lymphoma in the liver explant of a patient with MASH cirrhosis who underwent OLT. To our knowledge, the combination of low-grade hepatic lymphoma in MASH cirrhosis is unique in the literature. Undiagnosed incidental hepatocellular carcinoma is the most commonly reported liver explant pathology in the current literature, but multiple other reports of incidental nonhepatic malignant explant pathologies have been reported in patients with varying etiologies of cirrhosis/hepatitis, including mucosa associated lymphoid tissue lymphoma,^6^ intravascular large B-cell lymphoma,^7^ diffuse large B-cell lymphoma, germinal center phenotype,^4^ intravascular lymphoma,^8^ T-cell rich large B-cell lymphoma,^9–12^ mixed cellularity Hodgkin lymphoma,^13^ other lymphomas,^14,15^ and hepatocellular carcinoma^16^ (see Table 1 for details). Unlike our patient, several cases with lymphoma discovered on explant pathology were also worked up to have lymphoma, and most cases did require chemotherapy. However, many tolerated chemotherapy well and achieved remission post-OLT, suggesting that positive clinical outcomes were attainable while undergoing treatment for malignancy in the setting of a new transplanted liver. There has only been 1 similar case to our patient of a non-Hodgkin B-cell lymphoma reported in the explanted liver of a patient with MASH cirrhosis after OLT,^17^ and both patients remained asymptomatic without needing chemotherapy or radiation.

**Table 1.** Details of nonhepatic malignancies reported on explant pathology in patients with liver transplantation

Study	Pre-OLT diagnosis	Explant pathology	Workup post-explant pathology diagnosis	Malignancy treatment	Clinical outcomes
Orrego et al (2005)^6^	HCV cirrhosis	B-cell non-Hodgkin lymphoma of MALT type	No lymphadenopathy noted in pretransplant workup. CTAP postexplant negative for active disease; bone marrow biopsy post-OLT demonstrated lymphoma involvement	Completed 4-dose rituximab course	Tolerated chemotherapy well; developed CMV infection after second dose, treated with valganciclovir
Roshal et al (2008)^7^	HCV cirrhosis	Intravascular large B-cell lymphoma	Found to have bilateral adrenal masses on PET scan 2 months after transplantation. Biopsy of adrenal masses showed diffuse large B-cell lymphoma	Rituximab, did not complete course	Started on rituximab, but died 3 months later from progressive hepatic failure
López et al (2014)^5^	Alcoholic cirrhosis	Hodgkin lymphoma with nodular regenerative hyperplasia	Bone marrow aspiration negative for lymphoproliferative process	Treated with ABVD, developed hyperbilirubinemia in the fourth cycle, so treatment is suspended	Follow-up bone marrow biopsy and myelograms 5 months postchemotherapy were negative for active lymphoma. Patient was lost to follow-up 10 months post-transplantation
Kuramitsu et al (2015)^8^	Fulminant hepatitis of unknown etiology	Intravascular B-cell lymphoma	Bone marrow biopsy demonstrated normoblastic marrow with slight myelofibrosis and lymphocytes	Treated with 8 cycles of chemotherapy including R-CHOP	Remains free of recurrence 1 year post-transplantation
Cameron et al (2005)^9^	Fulminant hepatic failure of unknown etiology	Malignant T-cell rich, large B-cell non-Hodgkin lymphoma with widespread hepatocellular necrosis	No lymphadenopathy noted in pretransplant CT scans. Post-OLT bone marrow biopsy was negative. Whole-body PET scan showed increased activity in spleen suspicious for lymphoma	R-CHOP, specific regimen unspecified	Good clinical recovery post-OLT. No evidence of disease to date. Remains on standard immunosuppressive therapy
Luu et al (2020)^10^	Fulminant hepatic failure of unknown etiology	T-cell lymphoma	No hypermetabolic lesions noted on PET scan	Completed chemotherapy (unknown regimen)	Remains in clinical remission on immunosuppressive regimen
Ruiz et al (2023)^13^	Fulminant hepatic failure of unknown etiology	Mixed cellularity Hodgkin disease	Lumbar puncture showed no evidence of infiltration	Treated with 6 cycles of chemotherapy with BEACOPP	Achieved complete remission in 11 months, disease free for 3 years to date
Murta et al (2022)^16^	Cryptogenic cirrhosis	Synchronous presence of hepatocellular carcinoma, cholangiocarcinoma, and plasmacytoma	None	None; observed w/ preserved liver function post-OLT	Remains free of recurrence at least 3 years 8 months after transplantation

ABVD, adriamycin, blemomycin, vinblastine, dacarbazine; BEACOPP, bleomycin, etoposide, adriamycin, cyclophosphamide, vincristine, procarbazine, and prednisone; CMV, cytomegalovirus; DLBCL, diffuse large B-cell lymphoma; HCV, hepatitis C virus; MALT, mucosa-associated lymphoid tissue; OLT, orthotopic liver transplant; PET, positron emission tomography; R-CHOP, rituximab–cyclophosphamide, hydroxydaunorubicin, Oncovin, prednisone.

To reduce the chances of recurrence of lymphoma, we switched our patient from a calcineurin inhibitor (tacrolimus) to an mTOR inhibitor (everolimus). In addition to known benefits of improving long-term renal function in OLT patients, mTOR inhibitors have been used for their antioncogenic effects in post-transplant patients.^18^ Preliminary studies on use of mTOR inhibitor have shown promise for their role in preventing de novo post-transplant malignancies, as well as treating existing malignancies.^19^

Our patient underwent extensive pretransplant evaluation for perihepatic and periportal lymphadenopathy, including a CT scan and fine-needle aspiration biopsy, without evidence of malignancy. The liver tumor board reviewed the case and deemed the workup sufficient, opting not to pursue further malignancy investigation. However, we recognize that opportunities to investigate the lymphadenopathy further via an incisional biopsy would have been valuable to facilitate earlier lymphoma detection. In future cases, we recommend that liver transplant centers conduct a thorough malignancy workup on suspicious findings such as lymphadenopathy to facilitate informed transplant eligibility decisions.

Regarding transplant eligibility, active extrahepatic malignancy and hepatic malignancy with macrovascular or diffuse tumor invasion are absolute contraindications to OLT.^20^ However, given this patient's localized lymphadenopathy and lack of systemic spread or significant clinical signs of active malignancy, we suggest reconsidering extrahepatic malignancy as an absolute contraindication. Instead, it could be viewed as a relative contraindication, particularly given the patient's excellent post-OLT recovery despite malignancy noted on explant pathology. In future cases, we recommend a case-by-case evaluation of eligibility when extrahepatic malignancy is detected during pretransplant workup.

## DISCLOSURES

Author contributions: K. Mumtaz, H. Chi, and L. Kaufman started the first draft of manuscript. L. Bushrow and M. Yearsley provided the pathology images. All authors participated in finalizing the final draft of the case report. K. Mumtaz is the article guarantor.

Financial disclosure: None to report.

Previous presentation: This case was presented at the American College of Gastroenterology 2024 Annual Meeting; October 25-30, 2024; Philadelphia, PA.

Informed consent was obtained for this case report.
